# Health-related quality of life among community-dwelling people aged 80 years and over: a cross-sectional study in France

**DOI:** 10.1186/s12955-020-01376-2

**Published:** 2020-05-07

**Authors:** Isabelle Jalenques, Fabien Rondepierre, Chloé Rachez, Sophie Lauron, Candy Guiguet-Auclair

**Affiliations:** 1 Université Clermont Auvergne, CHU Clermont-Ferrand, Service de Psychiatrie de l’Adulte A et Psychologie Médicale, Centre Mémoire de Ressources et de Recherche, 58 rue Montalembert, Cedex 1, 63003 Clermont-Ferrand, France; 2 CHU Clermont-Ferrand, Service de Psychiatrie de l’Adulte A et Psychologie Médicale, Centre Mémoire de Ressources et de Recherche, Clermont-Ferrand, France; 3 CHU Clermont-Ferrand, Service de Psychiatrie de l’Adulte A et Psychologie Médicale, Clermont-Ferrand, France; 4 Université Clermont Auvergne, CHU Clermont-Ferrand, CNRS, SIGMA Clermont, Institut Pascal, Clermont-Ferrand, France

**Keywords:** Health-related quality of life, Elderly people, Older adults, Eighty and over, Ageing, Community-dwelling elderly people, LEIPAD

## Abstract

**Background:**

The proportion of people living to a very old age is continuously increasing. One of the possibilities explored in policies and services to meet this health and societal challenge is to encourage the very old to continue living at home. This initiative is in line with the wishes of most elderly people. However, owing to the great changes that occur during old age attention should be paid to health-related quality of life (HRQoL). The aims of this study were to assess HRQoL in French community-dwelling people aged 80 years and over and to investigate the sociodemographic and health characteristics and life events associated with HRQoL.

**Methods:**

A cross-sectional study was conducted in France to assess the HRQoL of people aged 80 years or more living at home. All people recruited were sent a letter explaining the aim of the study and requesting their consent to take part. Those who accepted then received a series of sociodemographic and medical questionnaires, a questionnaire concerning life events of the previous 12 months and the LEIPAD questionnaire, which assesses HRQoL in elderly people.

**Results:**

The data of 184 participants (54.9% female) with a mean age of 83.9 years (almost 40% older than 85 years), were analysed. Low scores, indicating better HRQoL, were obtained on the ‘Self-Care’ and ‘Depression and Anxiety’ scales with 50.9 and 40.8% of responders, respectively, having the minimum score of zero. The highest score was found on the ‘Sexual Functioning’ scale, with 59.1% of participants having the maximum score of 100. Elderly females declared a significantly less satisfactory HRQoL. Deteriorating health, an unsatisfactory environment, not being able to drive, perceived modest income and financial worries negatively affected HRQoL.

**Conclusion:**

Identifying factors in our study that are potential determinants of HRQoL would be of direct benefit for individuals. Concrete public policy initiatives concerning means of transport, living environment and financial resources could then be implemented to improve the HRQoL of very old community-dwelling individuals.

## Background

The populations of industrialised countries have seen a major increase in the last few decades in the number of people living to a very old age. At present, 5.3% of the population in Europe is aged 80 years and over [1] and this age group is currently the fastest growing on the continent and will continue to be so up to 2050 [2]. To meet the challenges that come with this trend, one of the areas of interest explored in policies and services is to encourage living in the community until very old age. This is in line with the wishes of most older people in Western countries, who prefer to live in their own familiar environment as long as possible rather than in institutions [3–5]. Such initiatives would also increase the ability of the health care systems to bear the financial costs [4]. However, owing to the great changes that occur in the long period of life that now extends beyond the age of 65, particularly in terms of health, particular attention needs to be paid to aged people’s quality of life (QoL). In 2014, total life expectancy at 65 years was 18.2 years, including 8.6 years with no activity restrictions for men, and 21.6 years, including 8.6 with no activity restrictions for women [6].

The quality of life is a broad concept covering all aspects of human activity. Health, the environment and social domain greatly influence the life of elderly people [4]. Health-related quality of life (HRQoL) is a reflection of the way that individuals perceive and react to their health-related factors, such as physical, functional, emotional, and mental well-being and to the nonmedical aspects of their lives such as family, friends and activities [7]. HRQoL and its determinants are therefore of interest to the individual from a medical and social point of view but also in shaping health policy [7]. Thus, measuring the HRQoL can be useful for studying some factors influencing housing decisions among elderly people and assessing how to act on these factors to promote the home support desired by the major part of elderly people and in the policies of many countries [5]. Measuring HRQoL of elderly people can also be useful when developing and then monitoring policy initiatives that are part of the dynamics of the Age-friendly Environments in Europe (AFEE) project [3]; for example by making it possible to assess the links between the physical or social environment and different dimensions of HRQoL. Precise and detailed HRQoL data from sufficiently large sample sizes of community-dwelling people aged 80 years are potentially of great interest but currently are scarce.

We assessed the HRQoL of community-dwelling elderly people (EP) aged 65 years and over in an earlier work [8] but, as in other studies, the number of participants aged 80 years and over was low (32 in our study) [9–11]. Among other studies, a Brazilian team investigated the quality of life of a community-dwelling population aged 60 years and over using the WHOQOL-BREF and the WHOQOL-Old. Of the 317 participants recruited 15.4% were octogenarians but the study gave no specific results for this subgroup [12]. In Europe, the very few recent studies involving community-dwelling people aged 80 years and over, all used the generic EuroQol five dimension scale (EQ-5D). One assessed people aged 75 years and over in six European countries using in addition the SF-12 questionnaire [13]; another, conducted in Switzerland, assessed people aged 65 years and over of whom 41% were 80 years old or more [14]; and a third assessed Dutch people aged 65 years and over of whom 11.8% were octogenarians [15]. In three of these studies, data on comorbidities were scarce or lacking. To our knowledge no recent study assessing HRQoL using an instrument specifically aimed at EP has been made in European community-dwelling people aged 80 years and over.

To assess HRQoL in older people, specific instruments, which are more suitable than generic ones because of their item relevance and better acceptability, have been developed and provide particularly useful information. They allow an easy individual assessment of older people’s HRQoL and can be used for medical and psychosocial interventions, as described by Xiao-Jun Lin et al. [7] Some of them have shown particularly interesting psychometric properties [16]. They include the LEIPAD, an acronym derived from the first two of the three universities involved in its development, LEIden in the Netherlands and PADua in Italy, under the auspices of the European office of World Health Organization. The LEIPAD is a brief self-administered HRQoL questionnaire developed specifically for community-dwelling EP and validated for French speaking people aged 80 years and over [17, 18].

Thus, the aims of this original study were first to measure HRQoL in a larger sample of community-dwelling people aged 80 years or over using a self-administered questionnaire touching on various aspects of daily life specifically adapted to EP, the LEIPAD questionnaire, and second to study its association with sociodemographic and medical characteristics and life events of this age group.

## Methods

### Study design

This cross-sectional study was conducted in France to evaluate HRQoL in EP aged 80 years of age and over who were not residents of an institution. The participants were picked at random (simple random sampling using random number tables) from electoral lists provided by the municipalities of six town councils (rural, semi-urban and urban) in the region of Auvergne (central France). In all, 2064 people were eligible, of whom 1501 were randomly selected to take part in the study. The sample size was calculated for a previous publication validating the LEIPAD questionnaire [18].

All potential participants were sent a letter explaining the aim of the study and requesting them to give consent to take part. Those who accepted then received a set of questionnaires, a letter detailing the procedure to be followed and a prepaid envelope in which to return the questionnaires.

The project was approved by the French regional ethics committee ‘Comité d’Ethique des Centres d’Investigation Clinique de l’Inter-région Rhône-Alpes-Auvergne - CE-CIC Grenoble’ (IRB 00005921) and conducted according to the principles expressed in the Declaration of Helsinki. All participants enrolled gave their written informed consent.

### Participants

To be included the participants had to be aged 80 years or over, living at home, not suffering from dementia or any other neurodegenerative disorder and capable of completing the self-report questionnaires without assistance.

Once all the data had been collected, we excluded from analysis participants who had not replied to the questionnaire unaided (identified by an item about whether they completed the questionnaires alone or with help) and those suspected of having a neurodegenerative disorder (identified by two items concerning health problems and current medical treatment).

### Data collection

Sociodemographic characteristics of the EP (gender, age, marital status, educational level, living status, ability to drive, hospital admission during the previous 12 months), their current health problems according to the ICD-10 classification, treatment being taken during the study, and events that may have disrupted their life in the previous 12 months (illness, poor health, daily care of a relative or friend, problem(s) with spouse or partner, bereavement, serious illness of a relative or friend, problem(s) with offspring, relational problem(s) with friends or relatives, moving house, environment perceived as unsatisfactory, income perceived as modest and financial worries were self-reported by participants and recorded. The variables analyzed were those that were studied and that had an impact on the HRQoL of community-dwelling elderly people aged 65 years and over [5, 19].

HRQoL was assessed by the LEIPAD questionnaire, a brief self-administered questionnaire for community-dwelling people aged 65 years and over [17] that allows an easy individual assessment of older people’s HRQoL and can be used for medical and psychosocial interventions [17]. The questionnaire is composed of 31 items grouped into seven scales: ‘Physical Function’ (5 items), ‘Self-Care’ (6 items), ‘Depression and Anxiety’ (4 items), ‘Cognitive Functioning’ (5 items), ‘Social Functioning’ (3 items), ‘Sexual Functioning’ (2 items) and ‘Life Satisfaction’ (6 items). All items are rated on a 4-point Likert scale, going from 0 (best HRQoL) to 3 (worst). A total score is calculated for each scale by adding up the individual scores of the items (provided that answers are given to all the questions). The score is then converted on a linear scale from 0 to 100, with lower scores indicating a better HRQoL. Its psychometric qualities have been widely demonstrated [16–18, 20]. A French version of the questionnaire was validated by our team specifically for use among community–dwelling people aged 80 years and above [18]. It showed very good acceptability, with response rates to each of the LEIPAD scales greater than 88%. Good internal consistency (Cronbach’s alpha ranging from 0.68 to 0.87) and strong test-retest reliability of the LEIPAD scales (intraclass correlation coefficients ranging from 0.77 to 0.95) were found.

### Statistical analyses

SAS v9.4 was used for all the statistical analyses. Statistical significance was set at *p*-value < 0.05.

Continuous data were expressed as the means and standard deviations (SD) or medians and interquartile range (IQR), and categorical data as frequencies and percentages.

Participants’ marital status, educational level, living status, ability to drive and hospital admission during the previous 12 months were compared according to gender and age by Chi-square tests and non-parametric Mann-Whitney tests, respectively. Current health problems according to the ICD-10 classification were compared according to gender by Chi-square tests or Fisher exact tests. The number of health problems was compared according to gender by non-parametric Mann-Whitney tests.

Bivariate associations between continuous scores on the LEIPAD scales (non-normal distributions) and participants’ characteristics (sociodemographic characteristics, self-reported health problems and life events in the previous 12 months) were analysed by non-parametric Mann-Whitney tests and Spearman’s correlation coefficients (to estimate correlations between age and scores). Multiple group comparison tests of the LEIPAD scales were performed with a Bonferroni correction, resulting in a corrected significant *p* value of *p* = 0.0073.

Owing to their asymmetric and non-normal distributions, the LEIPAD scores could not be included as dependent variables in multiple linear regression models. Some authors have dichotomized HRQoL scores according to the lowest quartile [21, 22] or according to the median [23, 24]. The quartiles have also been used to categorize HRQoL scores into four classes [24]. We did not dichotomize LEIPAD HRQoL scores because we would have discarded too much valuable information. We evaluated the categorization in four classes according to quartiles but there were insufficient numbers in each category. Hence, we chose an intermediate solution, and LEIPAD HRQoL scores were categorized into three ordinal classes designated as ‘poor’, ‘average’ and ‘good’ HRQoL, according to the first and third quartiles. The EP who had lower scores (lower than or equal to the first quartile) were considered to have ‘good’ quality of life, those having higher scores (greater than or equal to the third quartile) to have ‘poor’ quality of life, and those having intermediate scores (greater than the first quartile and lower than the third quartile) to have ‘average’ quality of life.

Bivariate ordinal logistic regressions were performed to assess the association between participants’ characteristics and LEIPAD scores categorized into the three ordinal classes. Crude odds ratios (OR), 95% confidence interval (CI) and its statistical significance were estimated. Finally, factors associated with HRQoL scores in the bivariate logistic regressions (found to be significant at the *p*-value level of 0.20 [25]) were included in multivariate ordinal logistic regression models using a forward selection, adjusting for gender and age. Adjusted odds ratio (AOR), 95% CI and its statistical significance were estimated. Cumulative logit models were used to study associations between factors and poorer quality of life. The parallel-lines model assumptions were verified. If the cumulative logit model was rejected, the adjacent categories model was used.

## Results

### Description of respondents

**Fig. 1:**
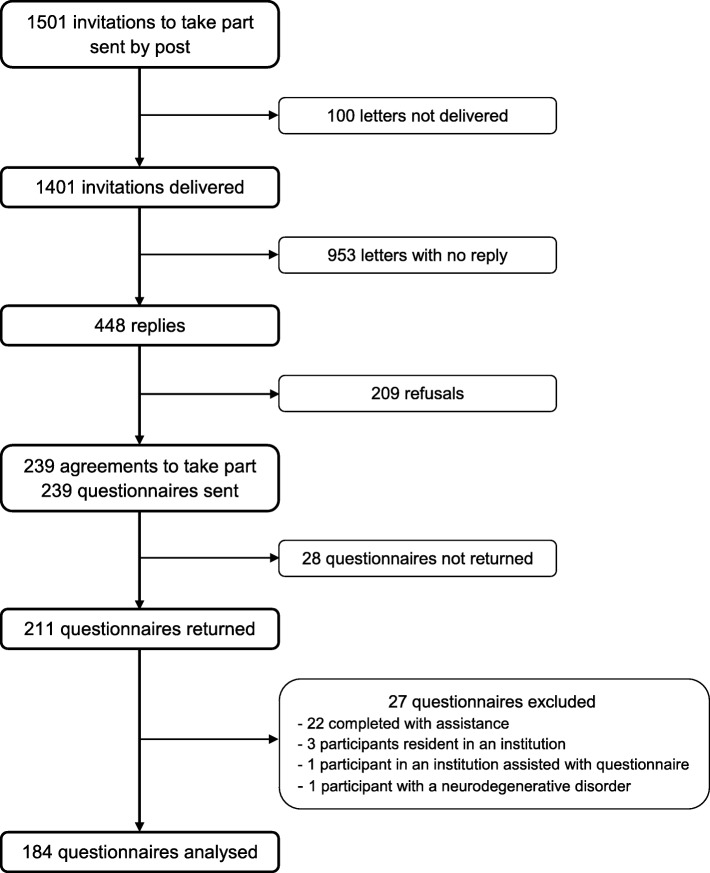
Organisation chart of participation in the study [18]. Of the 1501 people invited to take part, 448 (32%) replied to the request (100 letters were not delivered) with 239 accepting (53.3%) and 209 declining (46.7%). Of the 239 questionnaires sent to those who accepted to take part, 211 (88.3%) were returned. Of the latter, 27 were then excluded because they did not fulfil the inclusion criteria, leaving therefore 184 questionnaires to be analysed

The participation of EP in the study is described in Fig. 1.

The sociodemographic characteristics of the 184 EP recruited according to gender are given in Table 1. The participants were aged between 80 and 95 years: 61.4% between 80 and 84 years, 30.4% between 85 and 89, and 8.2% between 90 and 95. They were 2.8% never married, 58.6% married or living with a partner, 36.5% widowed and 2.2% divorced. The women were older than the men, were more often widowed, more often living alone and less likely to be driving. Those who were still driving were younger (83.2 years [SD 2.7] vs. 85.0 years [SD 3.8], *p* = 0.0009). The rate of hospital admission in the previous 12 months was 26.1% in those aged 80–84 years, 19.2% in those aged 85–89 years and 13.3% in those aged 90–95 years. It did not differ according to the participants’ gender (*p* = 0.1017) or age (*p* = 0.2279).

**Table 1:** Characteristics of the respondents (*n* = 184)

	Female	Male	Total	
	*n (%)*	*n (%)*	*n (%)*	***p-value***
**Gender**				
Female			101 (54.9%)	
Male			83 (45.1%)	
**Age** (years)				
*mean (SD)*	84.5 (3.6)	83.2 (2.8)	83.9 (3.3)	***0.0134***
*min-max*	80–95	80–90	80–95	
**Marital status**				***< 0.0001***
Never-married, widowed, divorced	63 (64.3%)	12 (14.5%)	75 (41.4%)	
Married or living with a partner	35 (35.7%)	71 (85.5%)	106 (58.6%)	
**Educational level**				*0.6040*
Pre-high school	80 (80.0%)	63 (76.8%)	143 (78.6%)	
High school or higher	20 (20.0%)	19 (23.2%)	39 (21.4%)	
**Living arrangement**				***< 0.0001***
Alone	59 (58.4%)	16 (19.3%)	75 (40.8%)	
Not alone	42 (41.6%)	67 (80.7%)	109 (59.2%)	
**Driving a car**				***< 0.0001***
Yes	33 (34.0%)	76 (95.0%)	109 (61.6%)	
No	64 (66.0%)	4 (5.0%)	68 (38.4%)	
**Hospital admission during the previous 12 months**				*0.1017*
Yes	18 (18.4%)	23 (28.8%)	41 (23.0%)	
No	80 (81.6%)	57 (71.3%)	137 (77.0%)	
**Life events during the previous 12 months**				
Illness	27 (27.8%)	25 (31.3%)	52 (28.3%)	*0.6196*
Poor health	25 (26.6%)	20 (25.0%)	45 (24.5%)	*0.8107*
Daily care of a relative or friend	12 (12.9%)	16 (20.0%)	28 (15.2%)	*0.2064*
Problem(s) with spouse or partner	5 (5.4%)	6 (7.5%)	11 (6.0%)	*0.5682*
Bereavement	25 (26.9%)	15 (18.8%)	40 (21.7%)	*0.2059*
Serious illness of a relative or friend	23 (24.7%)	26 (32.5%)	49 (26.6%)	*0.2582*
Problem(s) with offspring	19 (20.4%)	8 (10.0%)	27 (14.7%)	*0.0595*
Relational problem(s) with friends or relatives	8 (8.6%)	2 (2.5%)	10 (5.4%)	*0.1091*
Unsatisfactory environment	6 (6.5%)	7 (8.8%)	13 (7.1%)	*0.5675*
Perceived modest income	37 (39.8%)	17 (21.3%)	54 (29.3%)	***0.0087***
Financial worries	10 (10.8%)	7 (8.8%)	17 (9.2%)	*0.6591*

Statistical significance is shown in bold type

The events that had most frequently disrupted the life of the participants in the 12 months before the study are described in Table 1. There was no difference between women and men, except for perceived modest income and no difference according to age.

The health problems self-reported by the participants according to the ICD-10 classification are given in Supplementary Table 1. Almost all participants (97.8%) mentioned at least one health problem, with 10 being the greatest number. The women reported significantly more health problems than the men. The number of health problems was not correlated with age (*r* = 0.06, *p* = 0.4034). The most commonly cited diseases were those of the circulatory system, followed by disorders of the musculoskeletal system and connective tissue, and endocrine, nutritional and metabolic disorders. In addition, almost half of the participants reported visual disturbances or impairment or hearing impairment or deafness. Women reported more disorders of the musculoskeletal system and connective tissue and mental and behavioural disorders. Age was not related to health problems, except for mental and behavioural disorders for which participants were older (84.7 years (SD 3.4) vs. 83.6 years (SD 3.3), *p* = 0.0243).

### Health-related quality of life

The mean HRQoL scores of the LEIPAD scales are given in Table 2. High scores reflect a poorer HRQoL.

**Table 2:** Comparison of scores for dimensions on the LEIPAD scale according to the characteristics of the participants

	Physical Function	Self-Care	Depression and Anxiety	Cognitive Functioning	Social Functioning	Sexual Functioning	Life Satisfaction
**Total**	33.3 (20.0–46.7)	0 (0–22.2)	8.3 (0–25.0)	20.0 (6.7–26.7)	22.2 (11.1–33.3)	100 (83.3–100)	33.3 (22.2–38.9)
**Gender**							
Male	26.7 (13.3–40.0)	0 (0–5.6)	0 (0–16.7)	20.0 (6.7–26.7)	22.2 (11.1–33.3)	83.3 (66.7–100)	33.3 (22.2–38.9)
Female	33.3 (26.7–53.3)	11.1 (0–27.8)	16.7 (0–25.0)	20.0 (13.3–26.7)	22.2 (11.1–33.3)	100 (83.3–100)	33.3 (27.8–44.4)
*p-value*	***< 0.0001***	***< 0.0001***	***0.0018***	*0.3122*	*0.5249*	***< 0.0001***	*0.3153*
**Age** (years)							
r	0.25	0.28	0.15	0.13	0.13	0.16	0.10
*p-value*	***0.0010***	***0.0002***	*0.0515*	*0.0902*	*0.0778*	*0.0394*	*0.1884*
**Marital status**							
Never married, widowed, divorced	33.3 (26.7–53.3)	5.6 (0–27.8)	16.7 (0–25.0)	20.0 (6.7–26.7)	22.2 (11.1–33.3)	100 (100–100)	33.3 (27.8–44.4)
Married or with a partner	33.3 (20.0–46.7)	0 (0–16.7)	8.3 (0–25.0)	20.0 (6.7–33.3)	22.2 (11.1–33.3)	83.3 (66.7–100)	27.8 (22.2–38.9)
*p-value*	*0.0755*	*0.1331*	*0.3615*	*0.4597*	*0.2758*	***0.0002***	*0.0934*
**Educational level**							
Pre-high school	33.3 (20.0–53.3)	5.6 (0–22.2)	8.3 (0–25.0)	20.0 (6.7–33.3)	22.2 (11.1–33.3)	100 (83.3–100)	33.3 (25.0–38.9)
High school or higher education	26.7 (20.0–40.0)	0 (0–11.1)	8.3 (0–16.7)	13.3 (6.7–26.7)	22.2 (0–33.3)	83.3 (66.7–100)	33.3 (22.2–44.4)
*p-value*	*0.0700*	*0.0775*	*0.4631*	*0.4462*	*0.1481*	*0.0111*	*0.9059*
**Living arrangement**							
Alone	33.3 (20.0–53.3)	5.6 (0–22.2)	16.7 (0–25.0)	20.0 (6.7–26.7)	22.2 (11.1–33.3)	100 (83.3–100)	33.3 (27.8–44.4)
Not alone	33.3 (20.0–46.7)	0 (0–16.7)	8.3 (0–25.0)	20.0 (6.7–33.3)	22.2 (11.1–33.3)	100 (66.7–100)	27.8 (22.2–38.9)
*p-value*	*0.2278*	*0.6906*	*0.4764*	*0.5017*	*0.1645*	***0.0096***	*0.0224*
**Driving a car**							
Yes	26.7 (20.0–40.0)	0 (0–5.6)	0 (0–16.7)	20.0 (6.7–26.7)	22.2 (11.1–33.3)	100 (66.7–100)	27.8 (22.2–38.9)
No	40.0 (26.7–60.0)	22.2 (2.8–33.3)	16.7 (0–33.3)	20.0 (13.3–33.3)	22.2 (11.1–44.4)	100 (83.3–100)	33.3 (27.8–50.0)
*p-value*	***< 0.0001***	***< 0.0001***	***0.0033***	*0.0559*	*0.0174*	***0.0024***	*0.0814*
**Hospital admission during the previous 12 months**							
Yes	46.7 (20.0–60.0)	5.6 (0–22.2)	16.7 (0–33.3)	20.0 (6.7–40.0)	22.2 (11.1–33.3)	100 (83.3–100)	33.3 (22.2–38.9)
No	33.3 (20.0–40.0)	0 (0–16.7)	8.3 (0–25.0)	20.0 (6.7–26.7)	22.2 (11.1–33.3)	100 (66.7–100)	33.3 (22.2–41.7)
*p-value*	*0.0312*	*0.4275*	*0.1987*	*0.5622*	*0.6219*	*0.7535*	*0.6690*
**Number of health problems**							
r	0.50	0.41	0.25	0.21	0.24	0.08	0.30
*p-value*	***< 0.0001***	***< 0.0001***	***0.0008***	***0.0050***	***0.0011***	*0.2933*	***< 0.0001***
**Life events during the previous 12 months**							
**Illness**							
Yes	46.7 (33.3–60.0)	11.1 (0–27.8)	16.7 (0–33.3)	20.0 (6.7–40.0)	22.2 (11.1–33.3)	100 (66.7–100)	33.3 (22.2–44.4)
No	26.7 (20.0–40.0)	0 (0–16.7)	8.3 (0–25.0)	20.0 (13.3–26.7)	22.2 (11.1–33.3)	100 (83.3–100)	33.3 (22.2–38.9)
*p-value*	***< 0.0001***	***0.0030***	***0.0013***	*0.1877*	*0.6222*	*0.9003*	*0.4011*
**Poor health**							
Yes	53.3 (40.0–66.7)	22.2 (11.1–44.4)	16.7 (8.3–37.5)	20.0 (13.3–40.0)	33.3 (22.2–55.6)	100 (66.7–100)	38.9 (27.8–55.6)
No	26.7 (20.0–33.3)	0 (0–5.6)	8.3 (0–16.7)	20.0 (6.7–26.7)	22.2 (11.1–33.3)	100 (83.3–100)	27.8 (22.2–38.9)
*p-value*	***< 0.0001***	***< 0.0001***	***< 0.0001***	*0.0134*	***0.0002***	*0.8515*	***0.0003***
**Daily care of a relative or friend**							
Yes	36.7 (26.7–53.3)	11.1 (0–22.2)	16.7 (4.2–41.7)	26.7 (20.0–43.3)	33.3 (11.1–50.0)	100 (83.3–100)	38.9 (27.8–50.0)
No	33.3 (20.0–46.7)	0 (0–16.7)	8.3 (0–16.7)	13.3 (6.7–26.7)	22.2 (11.1–33.3)	100 (66.7–100)	27.8 (22.2–38.9)
*p-value*	*0.1579*	*0.2598*	***0.0048***	***0.0006***	*0.1544*	*0.4544*	***0.0024***
**Problem(s) with spouse or partner**							
Yes	33.3 (20.0–40.0)	0 (0–5.6)	12.5 (0–16.7)	20.0 (20.0–26.7)	22.2 (11.1–33.3)	100 (83.3–100)	33.3 (27.8–44.4)
No	33.3 (20.0–46.7)	0 (0–22.2)	8.3 (0–25.0)	20.0 (6.7–33.3)	22.2 (11.1–33.3)	100 (83.3–100)	33.3 (22.2–38.9)
*p-value*	*0.9014*	*0.1837*	*0.7097*	*0.2291*	*0.4988*	*0.6643*	*0.2447*
**Bereavement**							
Yes	33.3 (26.7–53.3)	5.6 (0–22.2)	25.0 (8.3–33.3)	20.0 (6.7–33.3)	33.3 (11.1–44.4)	100 (83.3–100)	38.9 (27.8–50.0)
No	33.3 (20.0–46.7)	0 (0–16.7)	8.3 (0–16.7)	20.0 (6.7–26.7)	22.2 (11.1–33.3)	100 (75.0–100)	27.8 (22.2–38.9)
*p-value*	*0.0245*	*0.4799*	***< 0.0001***	*0.2856*	*0.0411*	*0.9075*	*0.0278*
**Serious illness of a relative or friend**							
Yes	33.3 (26.7–53.3)	0 (0–16.7)	16.7 (0–33.3)	20.0 (10.0–33.3)	22.2 (11.1–44.4)	100 (83.3–100)	38.9 (27.8–50.0)
No	33.3 (20.0–46.7)	0 (0–16.7)	8.3 (0–16.7)	20.0 (6.7–26.7)	22.2 (11.1–33.3)	100 (66.7–100)	27.8 (22.2–38.9)
*p-value*	*0.1379*	*0.7422*	***0.0029***	*0.1529*	*0.2481*	*0.3812*	***< 0.0001***
**Problems(s) with offspring**							
Yes	40.0 (26.7–60.0)	11.1 (0–33.3)	16.7 (8.3–25.0)	20.0 (6.7–33.3)	33.3 (11.1–44.4)	83.3 (66.7–100)	44.4 (27.8–55.6)
No	33.3 (20.0–46.7)	0 (0–16.7)	8.3 (0–25.0)	20.0 (6.7–26.7)	22.2 (11.1–33.3)	100 (83.3–100)	27.8 (22.2–38.9)
*p-value*	*0.0352*	*0.0157*	*0.0456*	*0.5022*	***0.0044***	*0.2698*	***0.0054***
**Relational problem(s) with friends or relatives**							
Yes	46.7 (33.3–60.0)	25.0 (11.1–38.9)	20.8 (8.3–50.0)	26.7 (20.0–66.7)	50.0 (44.4–55.6)	100 (83.3–100)	52.8 (27.8–58.3)
No	33.3 (20.0–46.7)	0 (0–16.7)	8.3 (0–25.0)	20.0 (6.7–26.7)	22.2 (11.1–33.3)	100 (83.3–100)	33.3 (22.2–38.9)
*p-value*	*0.0137*	***0.0012***	*0.0305*	*0.0894*	***0.0004***	*0.6544*	*0.0528*
**Unsatisfactory environment**							
Yes	40.0 (40.0–50.0)	11.1 (5.6–25.0)	16.7 (16.7–33.3)	33.3 (20.0–46.7)	44.4 (33.3–66.7)	100 (83.3–100)	55.6 (50.0–66.7)
No	33.3 (20.0–46.7)	0 (0–16.7)	8.3 (0–25.0)	20.0 (6.7–26.7)	22.2 (11.1–33.3)	100 (75.0–100)	30.6 (22.2–38.9)
*p-value*	*0.0108*	*0.0453*	***0.0048***	*0. 0115*	***0.0002***	*0.6255*	***0.0002***
**Perceived modest income**							
Yes	40.0 (26.7–60.0)	11.1 (0–27.8))	16.7 (0–33.3)	26.7 (13.3–46.7)	22.2 (11.1–33.3)	100 (83.3–100)	38.9 (27.8–44.4)
No	26.7 (20.0–40.0)	0 (0–11.1)	8.3 (0–16.7)	13.3 (6.7–26.7)	22.2 (11.1–33.3)	100 (66.7–100)	27.8 (22.2–38.9)
*p-value*	***< 0.0001***	***0.0024***	***0.0002***	***0.0036***	*0.0467*	*0.0667*	***0.0001***
**Financial worries**							
Yes	40.0 (26.7–53.3)	2.8 (0–22.2)	16.7 (8.3–16.7)	20.0 (13.3–40.0)	22.2 (11.1–44.4)	91.7 (75.0–100)	44.4 (33.3–55.6)
No	33.3 (20.0–46.7)	0 (0–16.7)	8.3 (0–25.0)	20.0 (6.7–26.7)	22.2 (11.1–33.3)	100 (83.3–100)	27.8 (22.2–38.9)
*p-value*	*0.1597*	*0.6878*	*0.1492*	*0.1479*	*0.2374*	*0.8699*	***0.0003***

High scores indicate a poorer quality of life

For age and number of health problems, Spearman’s correlation coefficients are given. For sex, marital status, educational level, driving a car, hospital admission and life events during the previous 12 months, medians (interquartile range) are given. Statistical significance (*p*-value< 0.0073) is shown in bold type

The highest score was found on the ‘Sexual Functioning’ scale, with 59.1% of participants having the maximum score of 100. The answers showed that 61.2% were not interested in sexuality at all, 82.7% had no sexual activity and 14.5% occasionally.

Low scores, indicating better HRQoL, were obtained on the ‘Self-Care’ and ‘Depression and Anxiety’ scales with 50.9 and 40.8% of responders, respectively, having the minimum score of zero. The good responses obtained on the ‘Self-Care’ scale were due to the extent of the participants’ autonomy since 85.1% were able to get dressed unaided, 98.9% to eat without help, 84.1% to take a bath or shower by themselves and 72.3% to go shopping alone. For the ‘Depression and Anxiety’ scale, 16.1% admitted to feeling anxious or very anxious and in 6.1% of cases the effect on them was strong or fairly strong. Of the 4.5% who felt depressed or very depressed, 5.1% considered the effects to be strong or fairly strong.

Overall, 92.3% of the participants were satisfied with their social contacts and relations with other people, 87.3% with the way in which they organised their free or leisure-time activities and 86.7% with their financial situation.

In comparison to their past life, 74.9% of responders were satisfied with their current circumstances. However, 54.4% considered the future would be worse or far worse, and for 57.5% of the EP the idea of the future prevented them from planning or achieving what they would like to do. The participants considered their general state of health to be good or very good (69.1%), average (28.7%) or bad (2.2%).

**Fig. 2:**
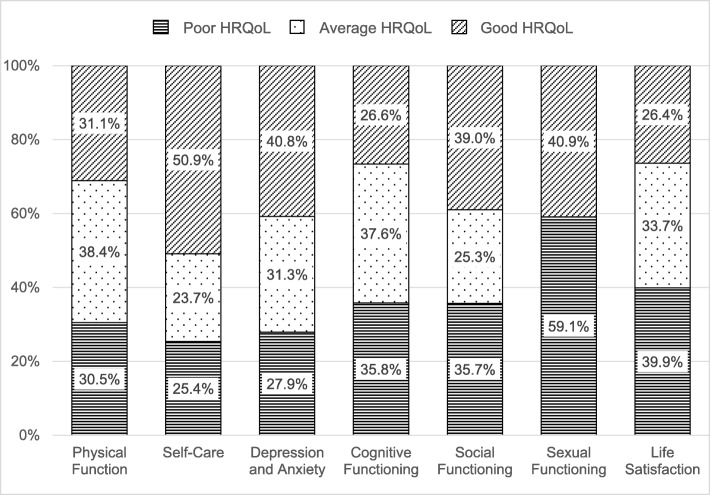
Distribution of the categorized LEIPAD scores. HRQoL: health-related quality of life

Distributions of the categorized LEIPAD scores into three ordinal classes according to the first and third quartiles are shown in Fig. 2. The prevalence of ‘poor’ quality of life was 30.5% for the ‘Physical Function’ scale, 25.4% for the ‘Self-Care’ scale, 27.9% for the ‘Depression and Anxiety’ scale, 35.8% for the ‘Cognitive Functioning’ scale, 35.7% for the ‘Social Functioning’ scale, 59.1% for the ‘Sexual Functioning’ scale and 39.9% for the ‘Life Satisfaction’ scale. The categorized score of ‘Sexual Functioning’ was binary (‘poor’ versus ‘good’).

### Risk factors for impaired health-related quality of life

The LEIPAD continuous scores of the EP were compared by bivariate analysis according to gender, age, marital status, educational level, ability to drive, and hospital admission, number of health problems and life events during the previous 12 months (Table 2).

Multivariate ordinal logistic regressions were then performed to explore the factors associated with poorer quality of life. LEIPAD scores categorized into three ordinal classes (‘poor’, ‘average’ or ‘good’ HRQoL) were used as dependent variables, and the significant factors in bivariate ordinal logistic regressions (*p* < 0.20) were covariates adjusted for gender and age. Marital status and living arrangement could not be added in regressions together because of collinearity. We conducted separate analysis with these variables. As the results were the same, we chose to present the models including marital status (which was also the most significant in models).

For the ‘Physical Function’ scale (Table 3), being a woman, older age, pre-high school educational level, greater number of health problems and self-reported poor health during the previous 12 months were associated with poorer quality of life. For the ‘Self-Care’ scale (Table 4), not driving, greater number of health problems and reported poor health during the previous 12 months negatively affected quality of life. For the ‘Depression and Anxiety’ scale (Table 5), female EP who reported poor health, bereavement, and serious illness of a relative or friend during the past year were more likely to have poorer quality of life. For the ‘Cognitive Functioning’ scale (Table 6), not being able to drive and providing daily care for a relative or friend during the previous 12 months were associated with poorer quality of life. For the ‘Social Functioning’ scale (Table 7), the risk of being in a lower category (and hence of having poorer quality of life) increased with older age, when participants were not able to drive a car, when they reported relational problems with friends or relatives and an unsatisfactory environment during the previous year. For the ‘Sexual Functioning’ scale (Table 8), never-married, widowed or divorced female EP with a pre-high school educational level were more likely to have poorer quality of life. Those who reported problems with offspring were more likely to have better quality of life in the sexual domain. For the ‘Life Satisfaction’ scale (Table 9), a serious illness of a relative or friend, unsatisfactory environment, perceived modest income and financial worries during the previous 12 months were associated with poorer quality of life.

**Table 3:** LEIPAD ‘Physical Function’ scale: ordinal logistic regressions (cumulative logit models)

		Bivariate ordinal logistic regression		Multivariate ordinal logistic regression ^a^	
	Prevalence of ‘poor’ HRQoL (%)	OR (95% CI)	*p-value*	AOR (95% CI)	*p-value*
**Gender**					
Male	17.7	Ref		Ref	
Female	40.8	2.78 (1.58–4.90)	***0.0004***	1.99 (1.02–3.89)	***0.0439***
**Age**					
(years)	84.9 (3.6) ^b^	1.10 (1.04–1.17)	***0.0019***	1.13 (1.02–1.26)	***0.0187***
**Marital status**					
Married or with a partner	27.2	Ref			
Never-married, widowed, divorced	33.3	1.58 (0.91–2.77)	*0.1076*		
**Educational level**					
High school or higher education	17.9	Ref		Ref	
Pre-high school	34.6	2.24 (1.14–4.37)	***0.0187***	2.62 (1.21–5.68)	***0.0147***
**Driving a car**					
Yes	17.3	Ref			
No	49.3	3.45 (1.90–6.27)	***< 0.0001***		
**Hospital admission during the previous 12 months**					
No	24.8	Ref			
Yes	51.3	1.66 (1.04–2.65)	***0.0354***		
**Number of health problems**	5.6 (1.9) ^b^	1.74 (1.47–2.07)	***< 0.0001***	1.63 (1.32–2.01)	***< 0.0001***
**Life events during the previous 12 months**					
**Illness**					
No	22.3	Ref			
Yes	51.0	3.38 (1.77–6.43)	***0.0002***		
**Poor health**					
No	15.4	Ref		Ref	
Yes	70.5	13.24 (6.03–29.05)	***< 0.0001***	9.22 (3.84–22.11)	***< 0.0001***
**Daily care of a relative or friend**					
No	28.3	Ref			
Yes	35.7	1.57 (0.74–3.33)	*0.2415*		
**Bereavement**					
No	27.3	Ref			
Yes	36.8	1.58 (0.81–3.10)	*0.1797*		
**Serious illness of a relative or friend**					
No	29.4	Ref			
Yes	29.8	1.30 (0.70–2.43)	*0.4045*		
**Problems(s) with offspring**					
No	27.3	Ref			
Yes	40.7	1.97 (0.91–4.25)	*0.0858*		
**Relational problem(s) with friends or relatives**					
No	27.6	Ref			
Yes	60.0	4.74 (1.28–17.49)	***0.0196***		
**Unsatisfactory environment**					
No	28.6	Ref			
Yes	41.7	2.32 (0.76–7.02)	*0.1381*		
**Perceived modest income**					
No	20.4	Ref			
Yes	49.1	3.47 (1.84–6.54)	***0.0001***		
**Financial worries**					
No	28.2	Ref			
Yes	41.2	1.94 (0.76–4.95)	*0.1678*		

Ordinal logistic regressions: (1) LEIPAD ‘Physical Function’ categorized score (‘poor’, ‘average’ or ‘good’ HRQoL) was used as the dependent variable. (2) Independent variables were entered as categorical variables except for age and number of health problems, which were entered as continuous variables. (3) *OR* odds ratio; *CI* confidence interval; *AOR* adjusted odds ratio

^a^ Multiple regression: adjusted for sex, age and for all variables included in the model

^b^ Mean (SD) in the ‘poor’ HRQoL category

Statistical significance is shown in bold type

**Table 4:** LEIPAD ‘Self-Care’ scale: ordinal logistic regressions (cumulative logit models)

		Bivariate ordinal logistic regression		Multivariate ordinal logistic regression ^a^	
	Prevalence of ‘poor’ HRQoL (%)	OR (95% CI)	*p-value*	AOR (95% CI)	*p-value*
**Gender**					
Male	10.3	Ref		Ref	
Female	37.9	4.01 (2.18–7.38)	***< 0.0001***	2.10 (0.82–5.38)	*0.1215*
**Age**					
(years)	85.4 (3.8) ^b^	1.12 (1.05–1.19)	***0.0002***	1.10 (0.98–1.23)	*0.1026*
**Marital status**					
Married or with a partner	21.8	Ref			
Never-married, widowed, divorced	29.6	1.61 (0.91–2.87)	*0.1033*		
**Educational level**					
High school or higher education	13.2	Ref			
Pre-high school	29.3	1.90 (0.93–3.89)	*0.0787*		
**Driving a car**					
Yes	8.7	Ref		Ref	
No	51.6	7.71 (4.03–14.78)	***< 0.0001***	4.58 (1.82–11.48)	***0.0012***
**Hospital admission during the previous 12 months**					
No	24.6	Ref			
Yes	31.6	1.38 (0.70–2.71)	*0.3533*		
**Number of health problems**	5.5 (2.1) ^b^	0.58 (1.33–1.87)	***< 0.0001***	1.37 (1.10–1.72)	***0.0057***
**Life events during the previous 12 months**					
**Illness**					
No	21.8	Ref			
Yes	34.7	2.17 (1.16–4.06)	***0.0159***		
**Poor health**					
No	12.3	Ref		Ref	
Yes	60.5	12.40 (5.83–26.38)	***< 0.0001***	12.61 (5.15–30.88)	***< 0.0001***
**Daily care of a relative or friend**					
No	24.1	Ref			
Yes	25.9	1.50 (0.69–3.25)	*0.3041*		
**Bereavement**					
No	23.6	Ref			
Yes	27.0	1.24 (0.62–2.47)	*0.5493*		
**Serious illness of a relative or friend**					
No	24.4	Ref			
Yes	24.4	0.76 (0.39–1.48)	*0.4185*		
**Problems(s) with offspring**					
No	22.3	Ref			
Yes	36.0	2.36 (1.06–5.22)	***0.0350***		
**Relational problem(s) with friends or relatives**					
No	22.1	Ref			
Yes	60.0	6.17 (1.69–22.59)	***0.0060***		
**Unsatisfactory environment**					
No	23.7	Ref			
Yes	33.3	2.37 (0.79–7.06)	*0.1230*		
**Perceived modest income**					
No	18.8	Ref			
Yes	36.5	2.61 (1.39–4.88)	***0.0028***		
**Financial worries**					
No	23.6	Ref			
Yes	31.3	1.26 (0.48–3.31)	*0.6458*		

Ordinal logistic regressions: (1) LEIPAD ‘Self-Care’ categorized score (‘poor’, ‘average’ or ‘good’ HRQoL) was used as the dependent variable. (2) Independent variables were entered as categorical variables, except for age and number of health problems which were entered as continuous variables. (3) *OR* odds ratio; *CI* confidence interval; *AOR* adjusted odds ratio

^a^ Multiple regression: adjusted for sex, age and for all variables included in the model

^b^ Mean (SD) in the ‘poor’ HRQoL category

Statistical significance is shown in bold type

**Table 5:** LEIPAD ‘Depression and Anxiety’ scale: ordinal logistic regressions (cumulative logit models)

		Bivariate ordinal logistic regression		Multivariate ordinal logistic regression ^**a**^	
	Prevalence of ‘poor’ HRQoL (%)	OR (95% CI)	*p-value*	AOR (95% CI)	*p-value*
**Gender**					
Male	22.0	Ref		Ref	
Female	33.0	2.44 (1.40–4.27)	***0.0018***	2.24 (1.20–4.16)	***0.0111***
**Age**					
(years)	84.4 (3.4) ^b^	1.06 (0.98–1.16)	*0.1349*	1.02 (0.93–1.11)	*0.7051*
**Marital status**					
Married or with a partner	28.8	Ref			
Never-married, widowed, divorced	27.0	1.27 (0.73–2.20)	*0.3939*		
**Educational level**					
High school or higher education	21.1	Ref			
Pre-high school	30.2	1.39 (0.71–2.71)	*0.3382*		
**Driving a car**					
Yes	21.5	Ref			
No	37.9	2.48 (1.39–4.42)	***0.0021***		
**Hospital admission during the previous 12 months**					
No	25.6	Ref			
Yes	36.6	1.47 (0.77–2.80)	*0.2460*		
**Number of health problems**	5.2 (2.0) ^b^	1.23 (1.07–1.41)	***0.0043***		
**Life events during the previous 12 months**					
**Illness**					
No	25.6	Ref			
Yes	35.3	1.90 (1.04–3.49)	***0.0383***		
**Poor health**					
No	21.6	Ref		Ref	
Yes	47.7	3.42 (1.77–6.61)	***0.0003***	2.56 (1.27–5.13)	***0.0082***
**Daily care of a relative or friend**					
No	24.3	Ref			
Yes	46.4	2.57 (1.20–5.51)	***0.0152***		
**Bereavement**					
No	20.9	Ref		*Ref*	
Yes	51.3	3.78 (1.89–7.55)	***0.0002***	*3.09 (1.48–6.42)*	***0.0026***
**Serious illness of a relative or friend**					
No	22.5	Ref		*Ref*	
Yes	41.7	2.41 (1.29–4.52)	***0.0060***	*1.97 (0.99–3.92)*	*0.0526*
**Problems(s) with offspring**					
No	27.5	Ref			
Yes	30.8	1.88 (0.87–4.08)	*0.1094*		
**Relational problem(s) with friends or relatives**					
No	26.6	Ref			
Yes	50.0	2.82 (0.84–9.46)	*0.0939*		
**Unsatisfactory environment**					
No	26.5	Ref			
Yes	46.2	3.25 (1.10–9.60)	***0.0333***		
**Perceived modest income**					
No	23.1	Ref			
Yes	39.2	2.24 (1.21–4.15)	***0.0100***		
**Financial worries**					
No	29.6	Ref			
Yes	12.5	1.12 (0.60–2.09)	*0.7260*		

Ordinal logistic regressions: (1) LEIPAD ‘Depression and Anxiety’ categorized score (‘poor’, ‘average’ or ‘good’ HRQoL) was used as the dependent variable. (2) Independent variables were entered as categorical variables, except for age and number of health problems which were entered as continuous variables. (3) *OR* odds ratio; *CI* confidence interval; *AOR* adjusted odds ratio

^**a**^ Multiple regression: adjusted for sex, age and for all variables included in the model

^b^ Mean (SD) in the ‘poor’ HRQoL category

Statistical significance is shown in bold type

**Table 6:** LEIPAD ‘Cognitive Functioning’ scale: ordinal logistic regressions (cumulative logit models)

		Bivariate ordinal logistic regression		Multivariate ordinal logistic regression ^a^	
	Prevalence of ‘poor’ HRQoL (%)	OR (95% CI)	*p-value*	AOR (95% CI)	*p-value*
**Gender**					
Male	33.3	Ref		Ref	
Female	38.0	1.41 (0.81–2.46)	*0.2202*	0.62 (0.29–1.33)	*0.2199*
**Age**					
(years)	84.5 (3.7) ^b^	1.10 (1.01–1.20)	***0.0237***	1.08 (0.99–1.19)	*0.0969*
**Marital status**					
Married or with a partner	37.9	Ref			
Never-married, widowed, divorced	32.4	0.89 (0.50–1.56)	*0.6764*		
**Educational level**					
High school or higher education	34.2	Ref			
Pre-high school	36.6	1.05 (0.54–2.03)	*0.8915*		
**Driving a car**					
Yes	29.1	Ref		Ref	
No	46.0	1.98 (1.10–3.58)	***0.0230***	2.59 (1.14–5.86)	***0.0226***
**Hospital admission during the previous 12 months**					
No	34.9	Ref			
Yes	41.0	1.17 (0.60–2.27)	*0.6401*		
**Number of health problems**	5.0 (1.8) ^b^	1.18 (1.02–1.36)	***0.0266***		
**Life events during the previous 12 months**					
**Illness**					
No	33.1	Ref			
Yes	44.9	1.53 (1.02–2.28)	***0.0397***		
**Poor health**					
No	32.0	Ref			
Yes	48.8	1.82 (0.95–3.50)	*0.0725*		
**Daily care of a relative or friend**					
No	32.1	Ref		Ref	
Yes	57.1	2.93 (1.32–6.51)	***0.0082***	3.30 (1.44–7.58)	***0.0049***
**Bereavement**					
No	32.8	Ref			
Yes	48.6	1.49 (0.75–2.95)	*0.2503*		
**Serious illness of a relative or friend**					
No	32.5	Ref			
Yes	45.8	1.47 (0.79–2.74)	*0.2292*		
**Problems(s) with offspring**					
No	34.5	Ref			
Yes	46.2	1.34 (0.61–2.91)	*0.4675*		
**Relational problem(s) with friends or relatives**					
No	35.3	Ref			
Yes	55.6	1.95 (0.54–7.03)	*0.3078*		
**Unsatisfactory environment**					
No	34.2	Ref			
Yes	61.5	3.25 (1.04–10.21)	***0.0431***		
**Perceived modest income**					
No	28.6	Ref			
Yes	52.8	1.71 (1.13–2.57)	***0.0106***		
**Financial worries**					
No	35.6	Ref			
Yes	43.8	1.67 (0.63–4.40)	*0.3025*		

Ordinal logistic regressions: (1) LEIPAD ‘Cognitive Functioning’ categorized score (‘poor’, ‘average’ or ‘good’ HRQoL) was used as the dependent variable. (2) Independent variables were entered as categorical variables, except for age and number of health problems which were entered as continuous variables. (3) *OR* odds ratio; *CI* confidence interval; *AOR* adjusted odds ratio

^a^ Multiple regression: adjusted for sex, age and for all variables included in the model

^b^ Mean (SD) in the ‘poor’ HRQoL category

Statistical significance is shown in bold type

**Table 7:** LEIPAD ‘Social Functioning’ scale: ordinal logistic regressions (adjacent categories models)

		Bivariate ordinal logistic regression		Multivariate ordinal logistic regression ^a^	
	Prevalence of ‘poor’ HRQoL (%)	OR (95% CI)	*p-value*	AOR (95% CI)	*p-value*
**Gender**					
Male	30.5	Ref		Ref	
Female	40.0	1.14 (0.81–1.60)	*0.4590*	0.61 (0.36–1.04)	*0.0709*
**Age**					
(years)	84.5 (3.3) ^b^	1.05 (1.00–1.11)	*0.0595*	1.07 (1.00–1.14)	***0.0439***
**Marital status**					
Married or with a partner	32.4	Ref			
Never-married, widowed, divorced	39.2	1.20 (0.85–1.70)	*0.2996*		
**Educational level**					
High school or higher education	30.8	Ref			
Pre-high school	37.3	1.30 (0.86–1.97)	*0.2168*		
**Driving a car**					
Yes	27.8	Ref		Ref	
No	47.8	1.57 (1.09–2.25)	***0.0147***	2.00 (1.14–3.51)	***0.0155***
**Hospital admission during the previous 12 months**					
Yes	31.7	Ref			
No	37.8	1.09 (0.73–1.64)	*0.6703*		
**Number of health problems**	4.9 (1.8) ^b^	1.14 (1.05–1.25)	***0.0037***		
**Life events during the previous 12 months**					
**Illness**					
Yes	32.7	Ref			
No	38.2	1.09 (0.75–1.59)	*0.6444*		
**Poor health**					
No	29.1	Ref			
Yes	57.8	1.92 (1.26–2.92)	***0.0026***		
**Daily care of a relative or friend**					
No	32.9	Ref			
Yes	53.6	1.29 (0.80–2.08)	*0.2996*		
**Bereavement**					
No	31.1	Ref			
Yes	53.8	1.34 (0.88–2.05)	*0.1726*		
**Serious illness of a relative or friend**					
No	34.4	Ref			
Yes	40.8	1.06 (0.72–1.56)	*0.7566*		
**Problems(s) with offspring**					
No	31.9	Ref			
Yes	59.3	1.79 (1.07–2.98)	***0.0261***		
**Relational problem(s) with friends or relatives**					
No	32.9	Ref		Ref	
Yes	90.0	5.28 (1.42–19.61)	***0.0129***	5.78 (1.46–22.91)	***0.0126***
**Unsatisfactory environment**					
No	32.3	Ref		Ref	
Yes	84.6	3.67 (1.43–9.43)	***0.0071***	3.87 (1.45–10.35)	***0.0070***
**Perceived modest income**					
No	33.1	Ref			
Yes	43.4	1.38 (0.94–2.03)	*0.0985*		
**Financial worries**					
No	35.7	Ref			
Yes	41.2	1.11 (0.62–2.00)	*0.7214*		

Ordinal logistic regressions: (1) LEIPAD ‘Social Functioning’ categorized score (‘poor’, ‘average’ or ‘good’ HRQoL) was used as the dependent variable. (2) Independent variables were entered as categorical variables, except for age and number of health problems which were entered as continuous variables. (3) *OR* odds ratio; *CI* confidence interval; *AOR* adjusted odds ratio

^a^ Multiple regression: adjusted for sex, age and for all variables included in the model

^b^ Mean (SD) in the ‘poor’ HRQoL category

Statistical significance is shown in bold type

**Table 8:** LEIPAD ‘Sexual Functioning’ scale: binary logistic regressions

		Bivariate logistic regression		Multivariate logistic regression ^a^	
	Prevalence of ‘poor’ HRQoL (%)	OR (95% CI)	*p-value*	AOR (95% CI)	*p-value*
**Gender**					
Male	43.8	Ref		Ref	
Female	71.9	3.29 (1.76–6.15)	***0.0002***	2.58 (1.7–5.69)	***0.0183***
**Age**					
(years)	84.3 (3.5) ^b^	1.10 (0.99–1.21)	*0.0612*	1.10 (0.97–1.25)	*0.1349*
**Marital status**					
Married or with a partner	47.6	Ref		Ref	
Never-married, widowed, divorced	76.1	3.50 (1.79–6.83)	***0.0002***	2.75 (1.19–6.32)	***0.0176***
**Educational level**					
High school or higher education	39.5	Ref		Ref	
Pre-high school	64.7	2.81 (1.34–5.89)	***0.0062***	2.92 (1.24–6.89)	***0.0141***
**Driving a car**					
Yes	50.9	Ref			
No	70.3	2.28 (1.18–4.40)	***0.0140***		
**Hospital admission during the previous 12 months**					
No	58.0	Ref			
Yes	57.5	0.98 (0.48–2.00)	*0.9539*		
**Number of health problems**	4.5 (2.0) ^b^	1.01 (0.87–1.18)	*0.8712*		
**Life events during the previous 12 months**					
**Illness**					
No	60.5	Ref			
Yes	54.0	0.77 (0.39–1.49)	*0.4339*		
**Poor health**					
No	59.3	Ref			
Yes	53.5	0.79 (0.39–1.59)	*0.5033*		
**Daily care of a relative or friend**					
No	57.2	Ref			
Yes	59.3	1.09 (0.47–2.51)	*0.8472*		
**Bereavement**					
No	58.6	Ref			
Yes	54.1	0.83 (0.40–1.74)	*0.6229*		
**Serious illness of a relative or friend**					
No	56.0	Ref			
Yes	61.2	1.24 (0.63–2.45)	*0.5380*		
**Problems(s) with offspring**					
No	60.1	Ref		Ref	
Yes	44.4	0.53 (0.23–1.22)	*0.1349*	0.29 (0.10–0.81)	***0.0186***
**Relational problem(s) with friends or relatives**					
No	57.4	Ref			
Yes	60.0	1.11 (0.30–4.10)	*0.8729*		
**Unsatisfactory environment**					
No	57.2	Ref			
Yes	61.5	1.20 (0.37–3.82)	*0.7635*		
**Perceived modest income**					
No	53.9	Ref			
Yes	66.0	1.66 (0.83–3.31)	*0.1506*		
**Financial worries**					
No	58.4	Ref			
Yes	50.0	0.71 (0.25–2.00)	*0.5203*		

Binary logistic regressions: (1) LEIPAD ‘Sexual Functioning’ categorized score (‘poor’ versus ‘good’ HRQoL) was used as the dependent variable. (2) Independent variables were entered as categorical variables, except for age and number of health problems which were entered as continuous variables. (3) *OR* odds ratio; *CI* confidence interval; *AOR* adjusted odds ratio

^a^ Multiple regression: adjusted for sex, age and for all variables included in the model

^b^ Mean (SD) in the ‘poor’ HRQoL category

Statistical significance is shown in bold type

**Table 9:** LEIPAD ‘Life Satisfaction’ scale: ordinal logistic regressions (cumulative logit models)

		Bivariate ordinal logistic regression		Multivariate ordinal logistic regression ^a^	
	Prevalence of ‘poor’ HRQoL (%)	OR (95% CI)	*p-value*	AOR (95% CI)	*p-value*
**Gender**					
Male	35.9	Ref		Ref	
Female	43.5	1.44 (0.82–2.55)	*0.2085*	1.32 (0.69–2.55)	*0.4032*
**Age**					
(years)	84.1 (3.3) ^b^	1.05 (0.96–1.14)	*0.2971*	1.02 (0.93–1.13)	*0.6389*
**Marital status**					
Married or with a partner	35.7	Ref			
Never-married, widowed, divorced	45.2	1.55 (0.86–2.80)	*0.1489*		
**Educational level**					
High school or higher education	47.1	Ref			
Pre-high school	38.3	0.98 (0.61–1.57)	*0.9269*		
**Driving a car**					
Yes	34.0	Ref			
No	49.1	1.68 (0.91–3.09)	*0.0952*		
**Hospital admission during the previous 12 months**					
No	41.9	Ref			
Yes	34.3	0.78 (0.39–1.57)	*0.4897*		
**Number of health problems**	5.0 (2.0) ^b^	1.29 (1.11–1.51)	***0.0013***		
**Life events during the previous 12 months**					
**Illness**					
No	41.6	Ref			
Yes	37.2	0.92 (0.48–1.75)	*0.7878*		
**Poor health**					
No	34.5	Ref			
Yes	57.9	2.67 (1.31–5.46)	***0.0072***		
**Daily care of a relative or friend**					
No	34.9	Ref			
Yes	63.0	3.20 (1.39–7.38)	***0.0065***		
**Bereavement**					
No	35.8	Ref			
Yes	54.5	1.90 (0.91–3.94)	*0.0873*		
**Serious illness of a relative or friend**					
No	30.5	Ref		Ref	
Yes	60.4	3.25 (1.66–6.37)	***0.0006***	3.78 (1.83–7.84)	***0.0003***
**Problems(s) with offspring**					
No	35.9	Ref			
Yes	63.6	2.99 (1.21–7.42)	***0.0179***		
**Relational problem(s) with friends or relatives**					
No	38.6	Ref			
Yes	62.5	2.64 (0.63–11.02)	*0.1843*		
**Unsatisfactory environment**					
No	36.6	Ref		Ref	
Yes	81.8	7.31 (1.57–33.95)	***0.0111***	7.36 (1.51–35.74)	***0.0134***
**Perceived modest income**					
No	31.1	Ref		Ref	
Yes	59.6	3.45 (1.75–6.81)	***0.0004***	2.46 (1.14–5.28)	***0.0211***
**Financial worries**					
No	36.2	Ref		Ref	
Yes	73.3	5.44 (1.62–18.21)	***0.0060***	5.49 (1.23–24.37)	***0.0253***

Ordinal logistic regressions: (1) LEIPAD ‘Life Satisfaction’ categorized score (‘poor’, ‘average’ or ‘good’ HRQoL) was used as the dependent variable. (2) Independent variables were entered as categorical variables, except for age and number of health problems which were entered as continuous variables. (3) *OR* odds ratio; *CI* confidence interval; *AOR* adjusted odds ratio

^a^ Multiple regression: adjusted for sex, age and for all variables included in the model

^b^ Mean (SD) in the ‘poor’ HRQoL category

Statistical significance is shown in bold type

## Discussion

This study makes two main contributions to the previous literature on HRQoL in EP. It is the first to assess the HRQoL of community-dwelling people aged 80 years and over (who are often few in number or absent from studies or whose age group is not studied specifically) with the use of a self-administered questionnaire specifically adapted to EP, the LEIPAD scale. In addition, it deals in detail with the comorbidities and life events of this age group and their relation to HRQoL.

Our study was guided by several important methodological aspects.

- HRQoL was assessed with a questionnaire touching on various aspects of daily life specifically aimed at EP. It has been validated in French for use among different sample populations including community–dwelling people aged 80 years and above, and its psychometric qualities, notably construct validity, internal consistency and reproducibility, have been ascertained [16, 18, 20].
- Our sample size is to our knowledge, after a reference database search, the largest to be used in an European study specifically focused on community-dwelling people aged 80 years and over whose HRQoL was assessed by a questionnaire specially adapted to their age group.
- Our study population was comparable to those in other published reports about French EP with regard to sociodemographic characteristics (French sample of 168 EP in the ESEMeD study) [13], living status [26, 27], ability to drive [28, 29], autonomy [30] and medical characteristics [27] except for the rate of eye diseases according to the ICD 10 classification, which was lower than that recorded in the general population of the same age [31], possibly because participants unable to complete the questionnaires unaided were not included in our study.

Some key points emerge from our study and are discussed in detail below: the declared HRQoL of EP aged 80 years of age and over was good for most dimensions but in certain domains was beginning to deteriorate, markedly so for the ‘Sexual Functioning’ scale; the female EP declared a significantly less satisfactory HRQoL in some domains; and some life events, other than health problems, negatively affected the HRQoL of EP in this age group.

**The declared HRQoL of our study population was good for most dimensions but in certain domains was beginning to deteriorate.** The best HRQoL score was obtained on the ‘Self-Care’ scale. This is an interesting finding because previous studies [13–15] used EQ-5D, in which self-care has a significant ceiling effect [14, 32]. Our results are consistent with those of a French survey, Handicap Santé 2008 [33], that used other means of assessment and according to which life expectancy in France at 65 years with no restrictions of personal care activities was 15.6 years for men and 17.9 years for women. These concordant results can be explained by the fairly good autonomy of our community-dwelling participants, most of whom were able to eat meals, get dressed, take a bath or shower and do their shopping without assistance. Similar levels of autonomy were also observed in several French other surveys [27, 30, 34]. For the ‘Physical Function’ and the ‘Self-Care’ scales, a greater number of health problems and reported poor health during the previous 12 months negatively affected quality of life, which corroborates and complements previous data for these domains in studies of populations that were generally less elderly or that focused on specific diseases [11, 12, 35].

A large majority of the participants also had a good HRQoL score on the ‘Depression and Anxiety’ scale. The score was not correlated with age, as in five other studies [13–15, 36, 37]. Our results are in line with those observed in the dimension of anxiety and depression in the EQ-5D [13–15]. However, the LEIPAD scales give a more nuanced description of this domain in the very elderly owing to distinct assessments of anxiety and depression and to an appreciation at four levels of intensity not only of what the participants feel subjectively but also of the functional impact of these feelings on their lives, which is a key factor. Our results in this domain can be explained in part by the state of health and individual autonomy of the participants. The effects of health problems on depression and anxiety have been described in a younger age group [8]. We also observed that poor health, bereavement and the illness of a relative or friend are factors that negatively affect this domain of HRQoL in those aged 80 or over. Almost three quarters of our EP reported being satisfied with their current condition in comparison to their past life, feelings echoed in studies involving younger elderly participants [8, 38]. The fact that half of our respondents were nevertheless pessimistic about their future can be explained by the strategy of “realistic expectations”: the greater the gap between one’s current situation and what one anticipates, the greater the risk of disappointment. To narrow this gap, the very elderly tend to revise their expectations downwards [39].

**The ‘Sexual Functioning’ scale had the poorest HRQoL scores in the present study.** Almost 60% of the respondents in the group had the highest score in this domain. However, this score did not significantly change as their age increased. Some studies have been made in the general population of the sexuality of EP up to the age of 78 years [40, 41] but to our knowledge only a very few studies of HRQoL have included the dimension of sexuality in the very elderly [42]. The response rate for items related to sexuality in our study was high (greater than 95%). In study populations not older than 65 years, the rate is generally far lower. A response rate similar to ours was, however, obtained by Molzhan et al. [42], who reported only 8.7% missing data for items related to sexual activity. The correlations evidenced in our study are consistent with the following explanatory hypotheses, which can of course be combined and are not the only ones possible.

- The fact that there were more women among the participants and that they reported a significantly poorer HRQoL for the dimension concerned with sexual activity is in agreement with recent studies showing that sexuality differs according to gender [42],
- The marital status of people aged 80 years and over plays an important role. In our study only 58.6% of the participants were married or living with a partner, and “having or not having a partner is the best predictor of sexual activity, especially for elderly women” [43, 44].

**The female EP in our study declared a significantly less satisfactory HRQoL** on the ‘Physical Function’, ‘Depression and Anxiety’ and ‘Sexual Functioning’ scales in multivariate analysis, which confirms and complements previous findings for these domains in populations that were generally less elderly or whose quality of life was assessed by generic questionnaires [10, 13, 15, 45–49]. Our findings also showed that the female participants had specific problems related to ageing that could in part explain their less satisfactory HRQoL: they were older than the men, more often widowed and living alone, had a perceived modest income, were less likely to be driving and were suffering from significantly more health problems, as reported in previous studies [27, 31, 37], in particular diseases of the musculoskeletal system and connective tissue and mental and behavioural disorders [50, 51]. Our study refines the results presented by König, namely that female gender was associated with more problems in most of the EQ-5D dimensions [13]. In our previous study of EP aged 65 years and over [8], HRQoL did not differ significantly according to gender, apart from sexual activity, for which the women reported a less satisfactory HRQoL. It would appear, therefore, that differences between men and women in terms of HRQoL arise with advancing age, which is consistent with findings of the “Vie Quotidienne et Santé” (vqs) survey of 2014, which suggested that in participants aged 75 years or over the situation is unfavourable for women in terms of functional impairments and limitations [27, 30]. The female EP also have a lower quality of life for the 'Self-care' dimension and, in multivariate models, a greater risk compared to men of having a deteriorated quality of life in this dimension. This is consistent with the fact that the total life expectancy at 65 years is longer in women compared to men, but that some of these “extra” years are with restrictions of personal care activities [6, 33]. Measuring HRQoL can be useful for studying the impact of some health problems, in particular diseases of the musculoskeletal system and mental and behavioral disorders, but also the establishment of actions and their impact on HRQoL in the health fields (prevention, screening, diagnosis and assessment, medical and psychosocial interventions, rehabilitation), the physical environment (for example, spaces and buildings accessible and useable by people with impairments, adjustment and improvement of elderly people's home) and community and health services (for example, home care, support for informal care) [3, 5].

**We identified which particular domains of HRQoL in the very elderly, other than those related to health, are negatively affected by life events.** For example, an unsatisfactory environment had a negative effect on the ‘Social Functioning’ and ‘Life Satisfaction’ scales. Not being able to drive had a negative effect on the ‘Self-Care’, ‘Cognitive Functioning’ and ‘Social Functioning’ scales. Incomes perceived as modest and financial worries during the previous 12 months were associated with a negative effect on the ‘Life Satisfaction’ scale. These findings are in line with those reported for EP aged between 65 and 75 years in studies of QoL [52, 53] and HRQoL [8] and enrich those of a study based on the EQ-5D in EP aged 75 years and over [13]. These findings can be useful during policy initiatives to create better age-friendly environments, whether in the field of outdoor environments (for example, support for community interaction and personal independence, places for recreation, physical activity and other leisure activities) or the domain of the social participation (for example, supportive environments for social exchange in the community). These findings can also be useful during policy initiatives concerning the domain of transport and mobility (for example, public transport, on-demand services and other support to improve mobility) [3]. As findings differ from one country to another, it is interesting to have recent data gathered in France.

### Limitations

Of the people contacted to take part in our study, 14.1% responded favourably, a proportion close to that in other surveys of this type among the elderly [20]. The large dropout was certainly due to our recruitment method, as only letters were sent to participants, without phone or other face to face contacts to present the study. Our study have to be replicated in different settings and with a different method of participants’ approach. It is likely that in the group of non-respondents there was a higher percentage of people with poorer health. Additionally, we excluded from analysis participants who had not replied to the questionnaire unaided. Thus, our population sample, and hence the study findings, are limited because the volunteer participants were particularly health-conscious or healthy. However, 69.1% of the participants considered their overall state of health to be good or excellent, a result in line with the findings of the Drees survey on the state of health of the general population in France performed in 2012 and according to which 69% of EP aged 85 years or over rated their overall state of health as “good” [54], and with a Finnish self-rated health study performed in 2009 [55]. The number of participants considering their state of health to be bad was very slightly lower than that in the vqs survey of 2014, which however included EP living in sheltered housing [27]. Currently in France, EP with severe loss of autonomy are very often housed in institutions, especially when they suffer from comorbid cognitive disorders [56]. The degree of autonomy of our participants was close to that described in French EP living at home [30].

We considered that the participants who were suffering from mental and behavioural disorders according to the ICD-10 classification were in fact suffering from psychiatric and not cognitive disorders since their treatment consisted solely of psychotropic drugs. We did not assess the cognitive status of the participants before they replied to the questionnaire but we discarded the questionnaires of those receiving treatment generally prescribed for cognitive disorders and those who did not complete the questionnaire unaided.

For some variables, there are few patients but our statistical tests took into account the small numbers in categories/variables if necessary. Our multivariate models were controlled for convergence. However, results on these variables had to be confirmed in larger samples.

## Conclusion

Longer life expectancy presents numerous challenges, notably preserving the highest levels of HRQoL as long as possible. In our study, EP living at home who were aged 80 years and over reported good HRQoL in most domains except that of ‘Sexual Functioning’. Their HRQoL became worse when their health deteriorated. The female EP declared a significantly less satisfactory HRQoL. An unsatisfactory environment, not being able to drive, perceived modest income and financial worries negatively affected HRQoL. Identifying factors in our study that are potential determinants of HRQoL would be of direct benefit for individuals and could lead to concrete actions in public policy concerning health services (particularly home care, medical and psychosocial interventions, rehabilitation), means of transport (particularly public transport and on-demand services), living environment (particularly home improvement, spaces and buildings accessible and useable by elderly people including with impairments) and financial resources. Such initiatives would help improve the HRQoL of EP living at home until very old age.

## Supplementary information

**Additional file 1: Table S1**: Health problems self-reported by the participants according to the ICD-10 classification

## Data Availability

The datasets used during the current study are available from the corresponding author on reasonable request.
