# Supplementary material for: Health-related quality of life among community-dwelling people aged 80 years and over: a cross-sectional study in France
Source: Health Qual Life Outcomes. 2020 May 7;18:126. doi: 10.1186/s12955-020-01376-2 (PMC7206748; doi:10.1186/s12955-020-01376-2)
Supplement: Supplementary file 1 — Additional file 1: Table S1: Health problems self-reported by the participants according to the ICD-10 classification [file 12955_2020_1376_MOESM1_ESM.docx]

**Supplementary Table 1: Health problems self-reported by the participants according to the ICD-10 classification**

|  | **Female** | **Male** | **Total** |  |
| --- | --- | --- | --- | --- |
|  | *n (%)* | *n (%)* | *n (%)* | ***p-value*** |
| **Number of health problems** |  |  |  |  |
| *mean (SD)* | *4.8 (2.1)* | *4.0 (1.8)* | *4.4 (2.0)* | ***0.0073*** |
| *min-max* | *0 - 10* | *0 - 8* | *0 - 10* |  |
| Diseases of the circulatory system | 81 (80.2%) | 66 (79.5%) | 147 (79.9%) | *0.9088* |
| Diseases of the musculoskeletal system and connective tissue | 76 (75.2%) | 42 (50.6%) | 118 (64.1%) | ***0.0005*** |
| Endocrine, nutritional and metabolic diseases | 66 (65.3%) | 51 (61.4%) | 117 (63.6%) | *0.5843* |
| Diseases of the eye and adnexa | 53 (52.5%) | 36 (43.4%) | 89 (48.4%) | *0.2189* |
| Diseases of the ear and mastoid process | 47 (46.5%) | 37 (44.6%) | 84 (45.7%) | *0.7909* |
| Diseases of the digestive system | 38 (37.6%) | 26 (31.3%) | 64 (34.8%) | *0.3721* |
| Mental and behavioural disorders | 42 (41.6%) | 11 (13.3%) | 53 (28.8%) | ***<0.0001*** |
| Diseases of the genitourinary system | 22 (21.8%) | 25 (9.30.1%) | 47 (25.5%) | *0.1969* |
| Diseases of the nervous system | 25 (24.8%) | 12 (14.5%) | 37 (20.1%) | *0.0830* |
| Diseases of the respiratory system | 18 (17.8%) | 17 (20.5%) | 35 (19.0%) | *0.6473* |
| Diseases of the blood and blood-forming organs and certain disorders involving the immune mechanism | 10 (9.9%) | 4 (4.8%) | 14 (7.6%) | *0.1958* |
| Neoplasms | 3 (3.0%) | 2 (2.4%) | 5 (2.7%) | *1.0000* |
| Certain infectious and parasitic diseases | 3 (3.0%) | 1 (1.2%) | 4 (2.2%) | *0.6282* |
| Diseases of the skin and subcutaneous tissue | 1 (1.0%) | 0 | 1 (0.5%) | *1.0000* |

Statistical significance is shown in bold type.
